# LT-171-861, a novel FLT3 inhibitor, shows excellent preclinical efficacy for the treatment of FLT3 mutant acute myeloid leukemia

**DOI:** 10.7150/thno.46593

**Published:** 2021-01-01

**Authors:** Zhou Yu, Jiaying Du, Hui Hui, Shaoxin Kan, Tongxin Huo, Kai Zhao, Tao Wu, Qinglong Guo, Na Lu

**Affiliations:** State Key Laboratory of Natural Medicines, Jiangsu Key Laboratory of Carcinogenesis and Intervention, School of Basic Medicine and Clinical Pharmacy, China Pharmaceutical University, 24 Tongjiaxiang, Nanjing 210009, People's Republic of China.

**Keywords:** Acute myeloid leukemia, FLT3, inhibitor, ITD, resistance

## Abstract

**Rationale:** Acute myeloid leukemia (AML) is a common type of haematological malignancy. Several studies have shown that neoplasia in AML is enhanced by tyrosine kinase pathways. Recently, given that aberrant activation of Fms-like tyrosine receptor kinase 3 (FLT3) acts as a critical survival signal for cancer cells in 20‒30% patients with AML, inhibition of FLT3 may be a potential therapeutic strategy. Therefore, we identified LT-171-861, a novel kinase inhibitor with remarkable inhibitory activity against FLT3, in preclinical models of AML.

**Methods:** We determined the inhibitory effects of LT-171-861 *in vitro* using AML cell lines and transformed BaF3 cells. Target engagement assays were used to verify the interaction between LT-171-861 and FLT3. Finally, a subcutaneous model and a bone marrow engrafted model were used to evaluate the antitumor effects of LT‑171‑861* in vivo*.

**Results:** Our data demonstrated that LT-171-861 had high affinity for FLT3 protein. We also showed that LT-171-861 had an inhibitory effect on FLT3 mutants in cellular assays. Moreover, LT-171-861 had a growth-inhibitory effect on human AML cell lines harboring FLT3 internal tandem duplications (FLT3-ITD) such as FLT3-D835Y, FLT3‑ITD-N676D, FLT3-ITD-D835Y, FLT3-ITD-F691L, FLT3-ITD-Y842C and AML blasts from patients with FLT3-ITD. Furthermore, LT-171-861 showed potent antileukemic efficacy against AML cells. We also show the efficacy of LT‑171-861 in a subcutaneous implantation model and a bone marrow engrafted model* in vivo*, where administration of LT-171-861 led to almost complete tumor regression and increased survival.

**Conclusions:** Overall, this study not only identifies LT-171-861 as a potent FLT3 inhibitor, but also provides a rationale for the upcoming clinical trial of LT-171-861 in patients with AML and FLT3-ITD mutations.

## Introduction

Acute myelogenous leukemia (AML) is a malignant heterogeneous clonal disorder of hemopoietic progenitor cells, characterized by aberrant differentiation and proliferation 1, 2. Fms-like tyrosine receptor kinase 3 (FLT3) plays a crucial role in normal hematopoiesis and leukemogenesis and is expressed in both myeloid and B‑lymphoid lineages 3. In AML patients, mutation of the FLT3 gene is the most frequent genetic alteration featuring an internal tandem duplication (ITD) within the juxtamembrane domain and single-base mutations within the tyrosine kinase domain (TKD) 2, 4. Mutation of FLT3-ITD was first reported by Nakao and colleagues in 1996 5, and was subsequently confirmed by several groups 6, 7. Overall, 20‒30% of patients with AML have ITD mutations in the FTL3 gene, which are associated with inferior clinical prognosis. FLT3-TKD has been also detected in ~5% of patients with AML. These gain-of-function changes within FLT3 result in constitutive activation without stimulation of the FLT3 ligand (FL), and sustained activation of downstream effectors including STAT5, AKT and ERK 8. The standard of care has predominantly been 7+3 induction cytotoxic chemotherapy with cytarabine and daunorubicin; therefore, a more effective therapeutic strategy is required. Given the central role of aberrant FLT3 activation in the progression of AML, targeting FLT3 is a promising therapeutic option 9.

In the past decade, several receptor tyrosine kinase inhibitors (TKIs) targeting FLT3 have been developed for the treatment of patients with AML 10. Non-specific inhibitors that target multiple genes including FTL3 have also been developed; lestaurtinib (CEP-701) 11, midostaurin (PKC412) 12, sorafenib (BAY 439006) 13, 14, tandutinib (MLN-518) 14 and sunitinib (SU11248) 15. Highly specific FLT3 inhibitors include AC220 16, gilteritinib (ASP2215) 17, and crenolanib (CP-868-596) 18. Although many of these inhibitors have been evaluated in clinical trials like MLN-518, a novel type III receptor tyrosine kinases inhibitor designed to treat AML in combination with other chemotherapeutic drugs 19, 20; few FLT3 inhibitors have been approved for monotherapy, such as AC220. Importantly, clinical responses for first-generation FLT3 inhibitors have been limited due to multi‑kinase inhibition and the development of resistance. Recently, it has been shown that the addition of midostaurin, the first US FDA approved FLT3 inhibitor, to standard chemotherapy significantly improved overall survival in young adult patients with AML and FLT3 mutations 21. However, it is difficult to apply this combination to older AML patients or those with comorbidities 22. The 5-year survival rate in patients < 60 years of age is around 40%, while in older patients and more high-risk patients is < 10%. Since the first-generation of FLT3 inhibitors were not originally screened for sensitivity and selectivity towards the activated FLT3 kinase, new-generation FLT3 inhibitors are required. AC220 is a second-generation FLT3 inhibitor that has shown strong efficacy in AML patients with FLT3-ITD in clinical trials 23. However, many responding patients did not achieve complete remission (CR) due to strong suppression of normal hematopoietic stem/progenitor cells because of the KIT-inhibitory effect of AC220 24. Furthermore, another mutation (FLT3-F691L) was acquired in some patients 2. All available clinical results indicate that new inhibitors must be able to target FLT3-ITD and FLT3‑TKD mutations and must not lead to toxic myelosuppression.

Here, we identified LT-171-861 as a potent FLT3 inhibitor with prominent activity against FLT3 kinase that led to sustained inhibition of FLT3 phosphorylation. In BaF3 model cells, LT-171-861 inhibited clinically known TKD mutants of FLT3 that confer resistance to sorafenib and AC220. Administration of LT-171-861 led to nearly complete tumor regression without relapse in a mouse xenograft model and increased survival in a bone marrow engraftment model. Therefore, LT-171-861 is a promising drug candidate with strong therapeutic potential for the treatment of AML.

## Methods

### Compounds, cell lines and plasmids

LT-171-861 was originally designed and synthesized by China Pharmaceutical University. Sorafenib (Cat. #3009) and AC220 (Cat. #5793) were purchased from ApexBio Company (Houston, TX). The human leukemia cell lines MV4-11, MOLM13, and murine pro-B cell line BaF3 were purchased from Cobioer company (Nanjing, China). Human leukemia cell line THP-1, U937 and human marrow stromal cell line HS-5 were originally obtained from the Cell Bank of Shanghai Institute of Cell Biology (Shanghai, China). MV4-11 cells were cultured in Iscove's Modified Dulbecco's Medium (IMDM) (Grand Island, NY) containing 10% FBS (Grand Island, NY). MOLM13, THP-1, and U937 cells were cultured in RPMI 1640 medium (Grand Island, NY) with 10% FBS. Murine pre-B cell line BaF3 was cultured in RPMI 1640 (Grand Island, NY) containing 10% FBS and 3 ng/mL interleukin (IL)-3 (R&D, MN, USA). HEK‑293T and HS-5 cells were cultured in Dulbecco's modified Eagle's medium (DMEM) (Grand Island, NY) supplemented with 10% FBS. Plasmids for construction of transformed BaF3-FLT3-wt and BaF3-FLT3-ITD were obtained from Dr. Bailiang He from The University of Hong Kong. A site-directed mutagenesis kit (Vazyme Biotech Co., Ltd, Nanjing, China) was used to generate plasmids for the construction of transformed BaF3-FLT3-ITD-F691L, BaF3-FLT3-ITD-N676D, BaF3‑FLT3-ITD-Y842C and BaF3-FLT3-D835Y.

### Expression and purification of FLT3

The intracellular domain of FLT3 (residues 571-994) was cloned into pMal-c2X containing an N-terminal MBP-tag with a TEV protease cleavage site. The recombinant vector was transformed into E. *coli*. BL21. Expression and purification of recombinant FLT3 were performed as described previously 25-27.

### Target engagement analyses

For kinase inhibition assays, the inhibitory activity of LT-171-861 against 15 kinases was performed by Reaction Biology Corporation (PA, USA). For the protein-based biolayer interferometry (BLI) assay, binding interaction analyses between FLT3 and LT-171-861 were performed on the Octet RED96 (CA, USA). All studies were performed with the Super Streptavidin (SSA) biosensors (CA, USA). All binding experiments were performed according to the manufacturer's instructions. For the cellular thermal shift assay (CETSA), experiments were performed as described previously 28.

### Generation of stable cell lines expressing human FLT3 with different mutations

Lentivirus packaging and construction of transformed BaF3 cells were performed as described previously 29. FLT3 mutant cDNA constructs or the vector, were co‑transduced with packaging plasmids (pMD2.G and pSPAX2) into HEK-293T cells according to the manufacturer's instructions. Supernatants were collected and used to infect BaF3 cells, and transduced BaF3 cells were selected with puromycin (1 μg/ml) to obtain stably transformed cells.

### Western blot

Western blot analysis was performed as previously described 30. AML cell lines, BaF3 transformed cell lines and human primary cells were treated with the indicated concentration of FLT3 inhibitors for 2 h. To examine the phosphorylation levels of FLT3-Y591 (Cat. #3464s), p44/42-Thr202/Tyr204 (Cat. #4370), whole-cell lysates were subjected to immunoblotting with the corresponding primary antibodies from Cell Signaling Technology (MA, USA). Anti-STAT5 (Cat. #a7733), anti-STAT5-Y694 (Cat. #ap0070), anti-ERK1/2 (Cat. #a1013), anti-AKT (Cat. #a7270), anti-AKT-Ser473 (Cat. #ap0098), anti-Caspase 3 (Cat. #a11021), anti-PARP1 (Cat. #a0010), anti-Bcl2 (Cat. #a19693), anti-Mcl-1 (Cat. #a0434), anti-Pim2 (Cat. #a9230), anti-Caspase8 (Cat. #a19549) and anti-FLT3 (Cat. #a12437) were purchased from ABclonal Company (Wuhan, China).

### Evaluation of apoptosis

Cells were seeded at a density of 2×10^5^ cells per well and treated with the indicated concentrations of test inhibitor for 36 h at 37 °C. Flow cytometry was carried out using an Annexin V-FITC Apoptosis Detection Kit from Vazyme Biotech Co. Ltd. (Nanjing, China).

### Cell proliferation and drug combination analysis

For the proliferation assay, cells were seeded at a density of 0.8×10^4^ cells per well and treated with the indicated concentrations of test inhibitor for 72 h at 37 °C. The conditional medium (CM) was prepared from the supernatant of HS-5 cell culture for 5 days under normal culture conditions. At the indicated time point, cell viability was evaluated by MTT assay.

For drug combination analysis, MOLM13 and MV4-11 cells were seeded at the same density for cell proliferation assay and treated with a cocktail of cytarabine and LT-171-861. After 72 h, cell viability was monitored using an MTT assay. The Combined Index was calculated using CompuSyn 1.0.

### Pharmacodynamic (PD) and pharmacokinetic (PK) studies

#### PD study

An MV4-11 cell tumor model was established by subcutaneous injection. Six-week-old female BALB/c nude mice were used for the subcutaneous model. Mice bearing tumors (approximately 120 mm^3^) were treated with LT-171-861 (10 mg/kg) by intravenous injection. Mice were sacrificed and tumor tissues were freshly dissected, homogenized, and centrifuged at 16000 g for 20 min. The phosphorylation levels of FLT3 were measured by Western blot.

#### PK study

Six-week-old female BALB/c mice were divided into two groups, and LT-171-861 was administered through intravenous injection and oral administration, respectively. After dosing, blood samples were collected at indicated time points (15 min, 30 min, 45 min, 60 min, 90 min, 2 h, 4 h, 6 h, 8 h, 12 h, 24 h). Blood samples from each group were collected at each time point and the supernatant was collected and used for HPLC/LC-MAS analysis.

### *In vivo* study

#### Subcutaneous model

According to previously described 13, 31, BALB/c nude mice were subcutaneously inoculated with MV4-11 cells (5×10^6^) and MOLM13 cells (5×10^6^). LT-171-861 was administered through intravenous tail injection every two days for 24 days, and sorafenib was orally administered every day. Tumor volume and body weight were measured with the caliper twice weekly. Tumor volumes were calculated based on: tumor volume=length×width^2^/2.

#### Bone marrow engrafted model

Six-week-old female NOD-SCID mice were treated with sub-lethal dose radiation (1.8 Gy) before intravenous injection of MV4-11 cells (5×10^6^) or MOLM13 cells (5×10^6^). LT-171-861 and vehicle control were dosed every two days for 24 days, and sorafenib was administered orally every day. The survival rate was determined by observation when the animals demonstrated hind-limb paralysis and became moribund.

General tissue morphology was visualized using hematoxylin and eosin (H&E) staining. Cell proliferation and apoptosis were evaluated by Ki67 Cell Proliferation Test Kit (Nanjing, China) and TUNNEL FITC Apoptosis Detection Kit (Nanjing, China).

Animal studies and euthanasia were carried out in strict accordance with the recommendations in the Guide for the Care and Use of Laboratory Animals of the China Pharmaceutical University.

### Primary human peripheral blood mononuclear cell (PBMC) samples

Primary cells from AML patients were obtained from Gulou Hospital (Nanjing, China) and Affiliated Zhongda Hospital of Southeast University (Nanjing, China). Normal PBMCs from 5 healthy donors were obtained from Affiliated Zhongda Hospital of Southeast University (Nanjing, China). Institutional Review Board approval in line with the Declaration of Helsinki was obtained for this study. Informed consent was obtained from all patients. Only samples with over 70% viability were used for subsequent studies. Normal PBMCs and leukemic blasts were purified and harevested in mononuclear layer as described previously 32. Detailed information on each patient is documented in supplementary Table S2.

## Results

### LT-171-861 selectively inhibits the proliferation of mutant FLT3-expressing leukemia cells* in vitro*

**Figure 1:**
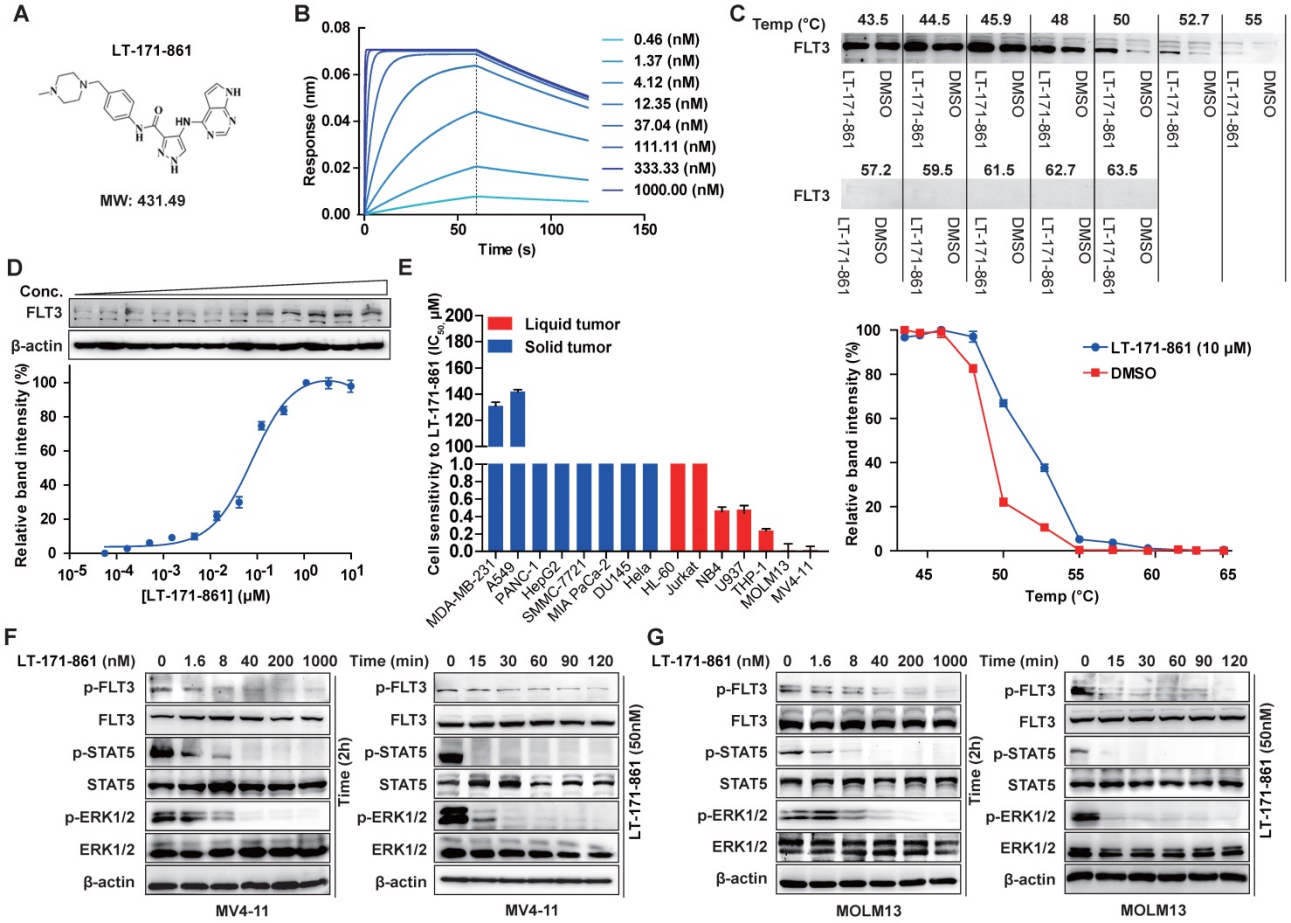
** The inhibitory efficacy of LT-171-861 against FLT3 kinase and mutant FLT3-expressing leukemia cell lines.** (**A**) Chemical structure of LT-171-861. (**B**) BLI assay was performed with 8 concentrations of LT-171-861. Data were then fitted to obtain K_D_ value. (**C, D**) Quantification was made using western blot (C: MV4-11 cells were treated with LT-171-861 (10 µM) for 1 h, and temperatures between 43.5-63.5 °C were defined to perform the test. D: MV4-11 cells were treated with LT-171-861 based on 11 different concentrations for 1 h and used to perform ITDR_CETSA_ assay at 53 °C) directed toward FLT3. Data were first normalized by setting the highest and lowest value in each set to 100% and 0% respectively. Data were obtained in the presence of the LT-171-861 (blue circle) as positive the control and DMSO (red square) as the negative control. All experiments were performed at three independent occasions and data are given as the mean ± SD. The solid lines represent the best fits of the data to the saturation binding curve model within the GraphPad Prism 5 software. (**E**) 8 solid tumor cell lines and 7 liquid tumor cell lines were incubated with increasing concentrations of LT-171-861 for 72 h. Cell viability was evaluated by MTT assay respectively. The IC_50_ values were calculated using nonlinear regression. (**F, G**) MV4-11 cells and MOLM13 cells were incubated with 50 nM of LT-171-861 respectively at indicated time points. MV4-11 cells and MOLM13 cells were incubated with the indicated concentrations of LT-171-861 for 2 h (right). p-FLT3 (Tyr 591), p-STAT5 (Tyr 694), p-ERK 1/2 (Tyr 204/Thr202) and each total protein were detected by western blot and β-actin was used as a loading control.

LT-171-861 is a novel small-molecule FLT3 kinase inhibitor designed and synthesized by China Pharmaceutical University (Figure 1A). Firstly, we characterized LT-171-861 with biophysical techniques to determine whether it could bind to the intended target. A BLI assay was used to test the affinity between FLT3 and LT-171-861. A concentration range from 0.3 nM to 1000 nM was defined (Figure 1B). In this assay, the K_D_ (3.31 nM) of LT-171-861 was calculated using a ForteBio Octet system., which indicated that LT-171-861 had high affinity to FLT3. CETSA has been used to identify FLT3-LT-171-861 interactions in live cell settings 28. We therefore applied this method to further confirm the interaction between FLT3 and LT-171-861 in our study (Figure 1C-D). We first verified that the inhibitor could stabilize FLT3 against denaturation using Western blot. We evaluated the thermal melt curves of FLT3 in the presence of LT-171-861 or dimethyl sulfoxide (DMSO). An obvious thermal shift of the melting curve was detected in the LT-171-861-treated sample (Figure 1C). Without LT-171-861, the apparent aggregation temperature (*T*_agg_) for FLT3 in MV4-11 cells was 49.11 °C. The *T_agg_* shifted to 52.91 °C in the presence of LT-171-861. Accordingly, we identified 53 °C as the optimal temperature for isothermal thermal concentration-response fingerprint (ITDRF) CETSA. The fitted K_d_ value was 78.48 nM for LT-171-861 (Figure 1D).

According to previous studies, major kinases inhibited by LT-171-861 were selected to determine their IC_50_ potency (Table 1). It has potent activity against FLT3, FLT3-D835Y and FLT3-ITD (IC_50_ was lower than 1 nM of each kinase). FLT4, FLT1, FGFR1, JAK3, JAK2, JAK1 and c-Src were also inhibited with an IC_50_ of 1‒70 nM. Other kinases including FMS, ERK2, c-Kit, PIM2 and ERK1, were inhibited to a lesser degree (IC_50_ > 200 nM).

Next, the inhibitory efficacy of LT-171-861 against FLT3 was confirmed in cell proliferation screening of tumor cell lines. LT-171-861 displayed higher inhibitory efficacy on FLT3-mutant cell lines than that on other tumor cell lines. The results of cell viability assays demonstrated that the proliferation of MV4-11 and MOLM13 cells were inhibited by LT-171-861 with IC_50_ values of 1.8 nM and 1.3nM, respectively (Figure 1E, Table 2). LT-171-861 had low inhibitory efficacy on cell lines expressing wild-type FLT3 (THP-1 and U937). To evaluate the inhibition efficacy of LT-171-861 against the autophosphorylation of FLT3, time-dependent and concentration-dependent experiments were used to challenge both MV4-11 and MOLM13 cells. LT-171-861 inhibited the autophosphorylation of FLT3 with an IC_50_ ≤40 nM, and the inhibition efficacy emerged rapidly after drug administration (Figure 1F-G).

In summary, LT-171-861 was identified as a novel and potent FLT3 inhibitor* in vitro* with high binding affinity for FLT3.

### LT-171-861 potently induces apoptosis of AML cell lines *in vitro*

**Figure 2:**
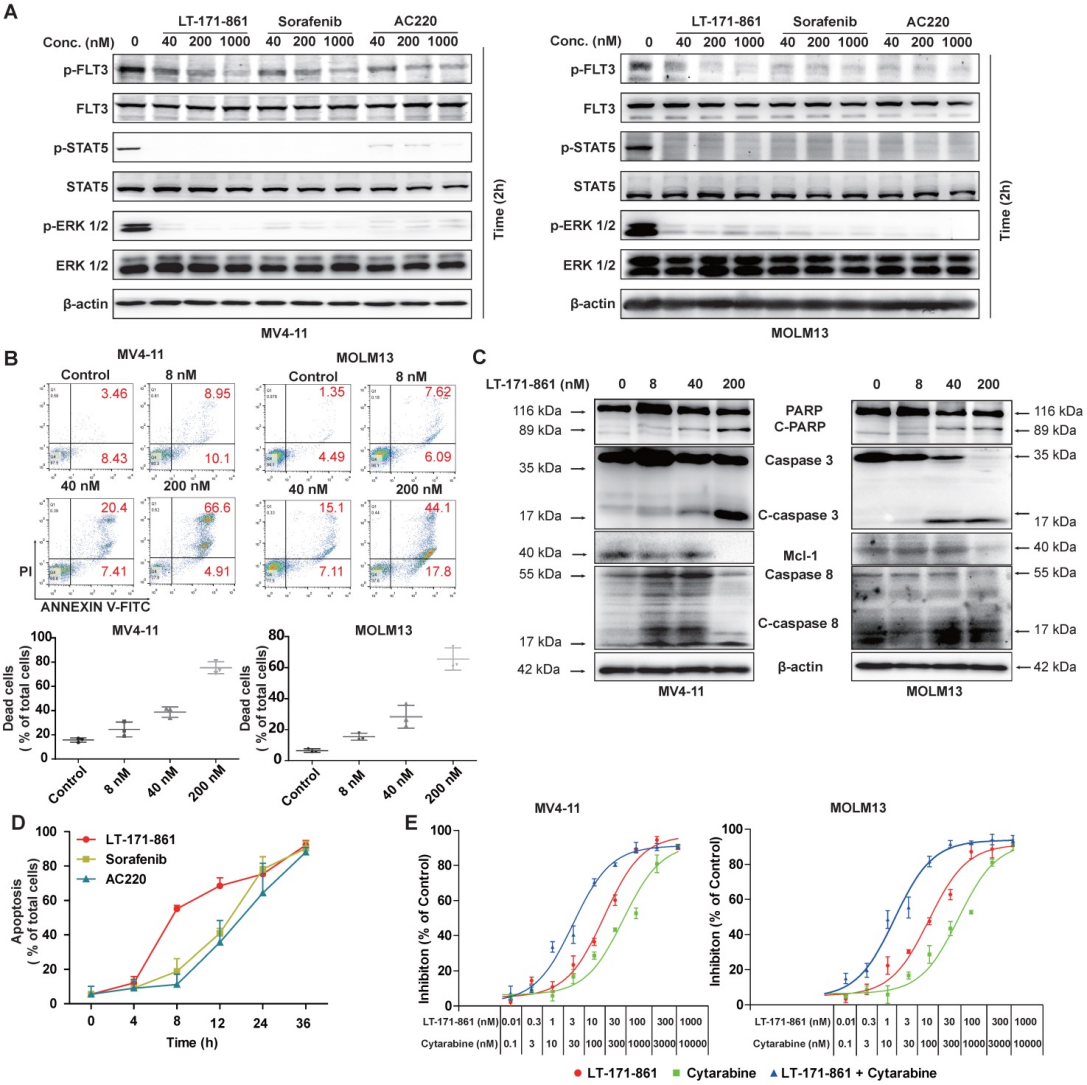
** Potent pro-apoptotic ability of LT-171-861 in leukemia cells harboring FLT3-ITD mutation.** (**A**) MOLM13 cells and MV4-11 cells were respectively incubated with indicated concentrations of LT-171-861, sorafenib and AC220 for 2 h. Cell lysates were used to perform western blot assay. (**B**) MV4-11 cells and MOLM13 cells were treated with LT-171-861 for 36 h. Cells then stained with propidium iodide (PI) /annexin V-FITC and then evaluated by flow cytometry. Annexin V^+^/PI^-^ and annexin V^+^/PI^+^ cells were considered dead cells and shown in the bottom chart (n=3). (**C**) MOLM13 cells and MV4-11 cells were treated with indicated concentrations of LT-171-861 for 36 h. PARP, caspase 3, caspase 8, Mcl-1 were evaluated in the cell lysate by western blot. (**D**) MV4-11 cells were treated with LT-171-861 (200 nM), AC220 (200nM) or sorafenib (200 nM) for indicated time periods (0, 4, 8, 12, 24, and 36 h) respectively. Then Cells were stained with propidium iodide/annexin V-FITC and evaluated by flow cytometry. (**E**) MOLM13 cells and MV4-11 cells were treated with cytarabine, LT-171-861 or a combination of cytarabine and LT-171-861 (ratio of 10:1), and cell viability was measured by MTT assay. Error bars show standard deviation (SD), and the combination index (CI) value was calculated by CompuSyn 1.0 software.

Previous studies have shown that auto-phosphorylation of FLT3 is observed when ITD mutation occurs in the juxtamembrane of FLT3 33. Therefore, inhibition of FLT3 and its downstream pathway is an important strategy. We used immunoblotting to detect the inhibition of FLT3 and its downstream pathway following treatment with LT-171-861, sorafenib and AC220 (Figure 2A). Our results showed that LT-171-861 inhibited FLT3 phosphorylation in a similar way to sorafenib and AC220.

Apoptosis assays were used to assess the pro-apoptotic ability of LT-171-861. Our data demonstrated that the treatment of LT-171-861 increased the apoptotic ratio of MV4-11 and MOLM13 cells bearing FLT3-ITD mutations (Figure 2B, Figure S1A). As shown in Figure 2C and Figure S1B, the proapoptotic proteins cleaved-caspase 3, cleaved-caspase 8, and PARP, as well as the antiapoptotic proteins Mcl-1 was measured by Western blot, demonstrating that LT-171-861 and established FLT3 inhibitors potently induced apoptosis in MV4-11 and MOLM13 cells. Next, we treated MV4-11 cells with LT-171-861, sorafenib and AC220 at indicated time points. A time-dependent experiment showed that LT-171-861 induced apoptosis in FLT3-ITD bearing cells more quickly than other established FLT3 inhibitors did (Figure 2D).

It has been proposed that the activity of FLT3 inhibitors increases synergistically when used in combination with cytarabine, a clinical chemotherapy drug. Therefore, we hypothesized that LT-171-861 may be useful in combination therapy (Figure 2E). The combination index (CI) value indicated that LT-171-861 had high synergistic anti‑proliferative activity at all concentrations tested (MV4-11 cell line: 50% effective dose, CI=0.056; 75% effective dose, CI=0.185; 90% effective dose, 0.640; MOLM13 cell line: 50% effective dose, CI=0.098; 75% effective dose, CI=0.288; 90% effective dose, CI=0.883), which demonstrates that LT-171-861 is suitable for combinational therapy (Figure S1C).

Altogether, these data indicate that LT-171-861 induces apoptosis rapidly and potently in FLT3-ITD-bearing cells, and has potential for combination therapy.

### LT-171-861 significantly inhibits FLT3 mutants in a transformed BaF3 cell model and FLT3 pathways in the stroma-protecting milieu

**Figure 3:**
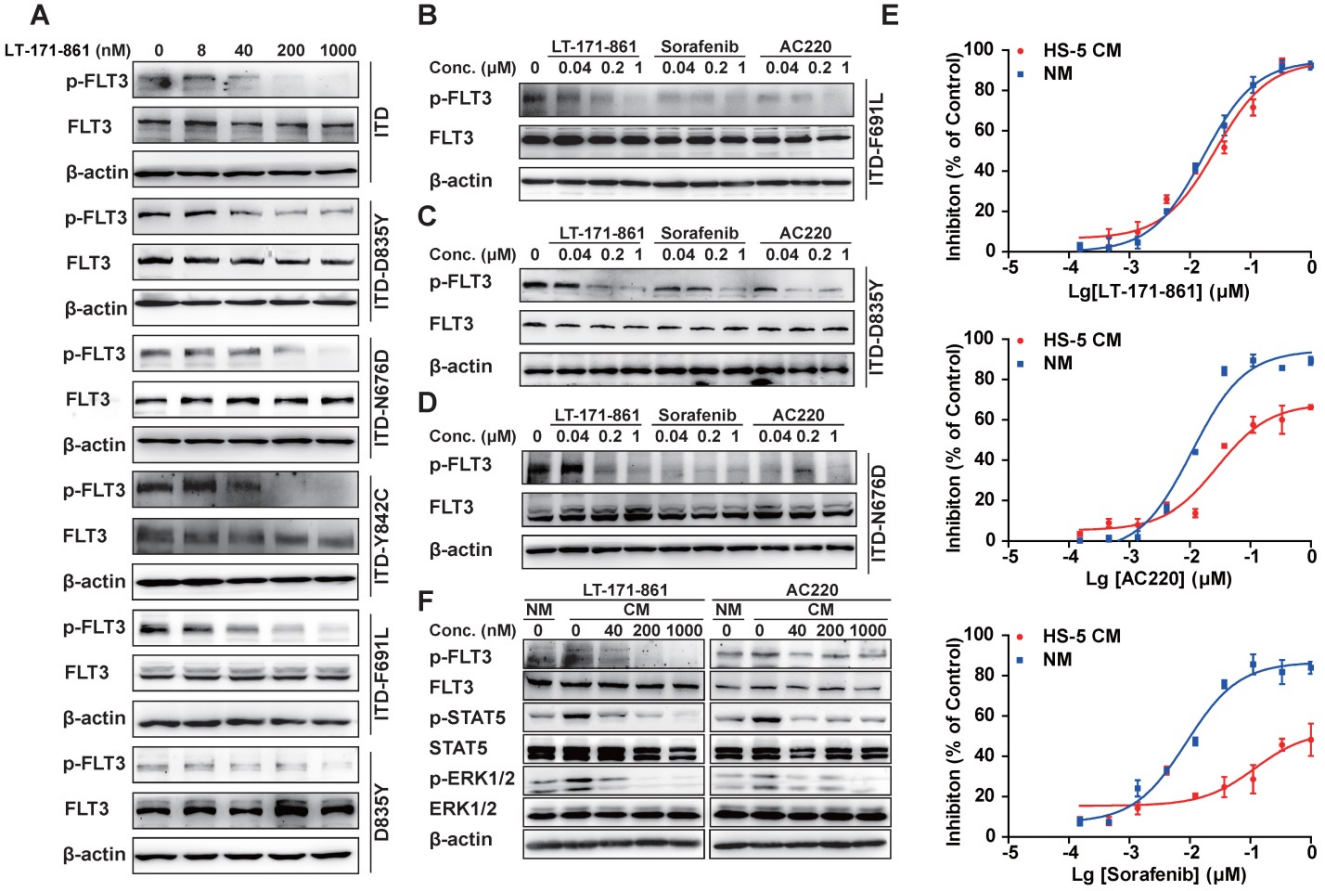
** Inhibition of FLT3 in BaF3 cell models and in stroma-protecting setting.** (**A**) Indicated FLT3 mutations were expressed in BaF3 cells respectively. Transformed BaF3 cells were incubated for 2 h with indicated concentrations of LT-171-861, and (**B-E**) for direct comparison with 3 inhibitors. The autophosphorylation level of FLT3 was visualized and compared by western blot analysis. (E) MOLM13 cells were treated with indicated concentrations of LT-171-861, sorafenib and AC220 for 72 h in condition medium (CM) and normal culture medium (NM). The CM was prepared from HS-5 cell culture for 4 days under routine culture conditions, clarified by centrifugation, and used immediately. The CM was added to complete medium at a final concentration of 40%. Cell viabilities were measured by MTT assay. Data were shown as mean±SD. (**F**) MOLM13 cells were incubated for 8 h in CM with indicated concentrations of LT-171-861 and AC220. The FLT3 phosphorylation levels of FLT3, STAT5 and ERK 1/2 were determined by Western blot.

TKD mutations are known to be an important cause of drug resistance in targeted therapy. To evaluate whether LT-171-861 is able to inhibit several FLT3 mutants that have been associated with drug resistance, we examined its potency against BaF3 cell lines that stably express FLT3-ITD, FLT-ITD/N676D, FLT3-ITD/F691L, FLT3-D835Y, FLT3‑ITD/D835Y and FLT3-ITD/Y842C. Among these mutants, N676D is an ATP-binding domain mutation, whilst both Y842C and D835Y belong to the activation loop, and F691L is a gatekeeper mutation. AC220 showed weaker inhibitory efficacy against BaF3 cells with FLT3-ITD/F691L mutations (IC_50_: 100.9 nM) and FLT3-ITD/N676D mutations (IC_50_: 212.4 nM), while sorafenib showed decreased potency against those expressing FLT3-ITD/D835Y mutations (IC_50_: 878.3 nM) and FLT3-D835Y mutations (IC_50_: 235.4 nM) compared with LT-171-861 (Table 2). In contrast, LT-171-861 showed stronger inhibitory activity against BaF3 cells expressing FLT3 with various mutations (ITD, ITD-D835Y, ITD-N676D, ITD-Y842C, ITD‑F691L and D835Y), and inhibitory levels of FLT3 phosphorylation were correlated with antiproliferation activities of LT-171-861 (Figure 3A; Table 2). Furthermore, we used BaF3 cells expressing FLT3-ITD/D835Y, FLT3-ITD/F691L, and FLT3-ITD/N676D to evaluate the inhibitory activities of three FLT3 inhibitors (Figure 3B-D). LT-171-861 inhibited FLT3 autophosphorylation at a lower concentration in FLT3-ITD/N676D cells compared with AC220, at a lower concentration in FLT3-ITD/D835Y cells compared with sorafenib, and at a comparable concentration in FLT3-ITD/F691L cells compared with AC220.

On the other hand, the bone marrow stroma-protecting environment containing a number of cytokines (e.g. FLT3 ligand, stromal derived factor-1, etc) could also decrease the drug efficacy of FLT3 inhibitors by activating FLT3 or its downstream effectors and building cross-talk between leukemic blasts and bone marrow microenvironment 34, 35. As previously described 31, 36, 37, we established a stroma-protecting model to evaluate whether LT-171-861 could resolve the problem. MOLM13 cells were cultured with CM or normal medium (NM) for 72 h (Figure 3E). LT-171-861 inhibited the proliferation of MOLM13 cells both in CM (24.7 nM in IC_50_) and NM (19.9 nM in IC_50_). However, CM impaired the inhibitory activities of AC220 (IC_50:_ 116.2 nM) and sorafenib (IC_50:_ 1.46 μM) compared with NM (AC220, IC_50:_ 14.12 nM in; sorafenib, IC_50:_ 11.34 nM). We also showed that LT-171-861 has comparable potency in CM to that of AC220 (Figure 3F), which may be attributed to its broad target profile, including JAK3, which is less sensitive to LT-171-861 treatment.

### PK/PD and toxicity profiling of LT-171-861

**Figure 4:**
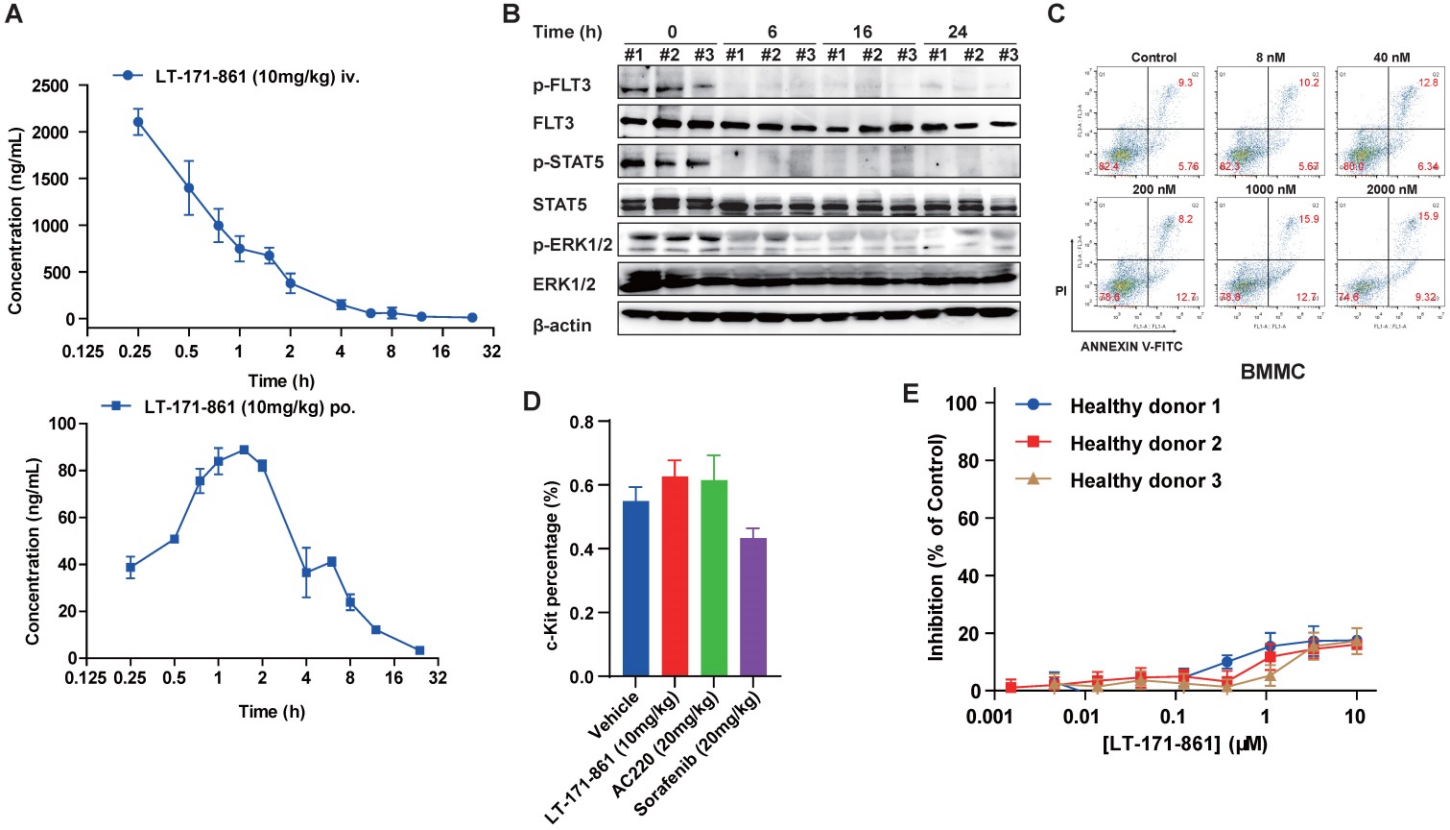
** PK/PD and preliminary toxicity evaluations of LT-171-861.** (**A**) Concentrations of LT-171-861 in plasma collected at indicated time points were measured by HPLC/LC-MAS analysis. Blue square and solid line represent intravenous injection administration. (**B**) PD study of LT-171-861 in BALB/c nude mice bearing tumors (120 mm^3^). Mice were sacrificed at indicated time points and then tumor tissues were dissected and lysed. Phosphorylation levels of FLT3, STAT5 and ERK1/2 were measured by western blotting. (**C**) Bone marrow mononuclear cells (BMMCs) were isolated from healthy C57BL/6 mice. Cells were treated with indicated concentrations of LT-171-861. Apoptosis assays were conducted after 36-hour incubation. (**D**) Healthy C57BL/6 mice (n=6) were intravenously injected with vehicle or LT-171-861 (10 mg/kg) every other day and were orally administered with AC220 (20 mg/kg) or sorafenib (20 mg/kg) once per day for 18 days. 24 h after the last administration, c-Kit^+^ cells were evaluated by flow cytometry. (**E**) PBMCs collected from 3 healthy donors, were incubated with LT-171-861 at indicated concentrations for 72 h and cell viabilities were conducted to evaluate by MTT assay. Data were shown with mean±SD.

To assess the PK properties of LT-171-861, a single dose of 10 mg/kg was administered to two independent groups of mice. Then, plasma levels of LT-171-861 were measured at each indicated time point. LT-171-861 was well absorbed and achieved the maximum plasma level (C_max_) of 50.06 μM (2167.50 ng/mL) within 3 h after intravenous injection and C_max_ of 0.20 μM (88.01 ng/mL) within 2 h after oral administration. The apparent plasma half-life was approximately 2 h for tail injection and 3.68 h for oral administration. Although the plasma half-life of LT-171-861 is limited, the plasma concentrations (i.v: 50.15nM and p.o: 30.00 nM) of LT-171-861 were above the IC_50_ of cellular assays 12 h after administration. After 12 h, the estimated free LT-171-861 remained at concentrations higher than the cellular IC_50_ in two drug‑dosing models (Figure 4A, supplementary table S1). The oral bioavailability of LT-171-861, determined in this experiment by comparing oral and intravenous pharmacokinetics at 10 mg/kg, was approximately 15%. To further understand the activity of LT-171-861, PD in tumor-bearing mice was investigated. A single dose of LT-171-861 (10 mg/kg) was administered to subcutaneous MV4-11-xenograft mice, which showed sustained inhibition of p-FLT3, p-STAT5 and p-ERK1/2 (Figure 4B), indicating that LT-171-861 effectively inhibited autophosphorylation of FLT3 and its downstream effectors. This inhibition efficacy lasted for 24 h, in accordance with the results of the PK experiment.

Furthermore, we preliminarily evaluated the toxicity profile of LT-171-861. As previously described 38, apoptosis assays were carried out to assess the effects of LT-171-861 on normal bone marrow mononuclear cells (BMMCs) isolated from healthy C57BL/6 mice. Cells were treated with LT-171-861 for 36 h, which led to hypotoxicity in normal BMMCs (Figure 4C). To further assess the potential toxicity of LT-171-861 in normal tissues (short-term toxicity assay), especially in hematopoietic cells, we treated normal C57Bl/6 mice with LT-171-861, AC220 or sorafenib for 18 days. Then, we evaluated progenitor cell (c-Kit^+^) lineages of bone marrow cells (Figure 4D), spleen or liver weights (Figure S2A), relative body weights (Figure S2B), and parameters of blood routine examination (Figure S2C). We also evaluated the potential toxic effects of LT-171-861 in human PBMCs obtained from 5 healthy donors (Figure 4D; Figure S2D). We found that the inhibition ratio of LT-171-861 to normal PBMC was 20% less than that of the control. Low anti-proliferation efficacy of LT-171-861 on normal PBMCs was observed in our study. These data demonstrated that LT-171-861 has acceptable toxicity.

### LT-171-861 is efficacious in an AML animal model

**Figure 5:**
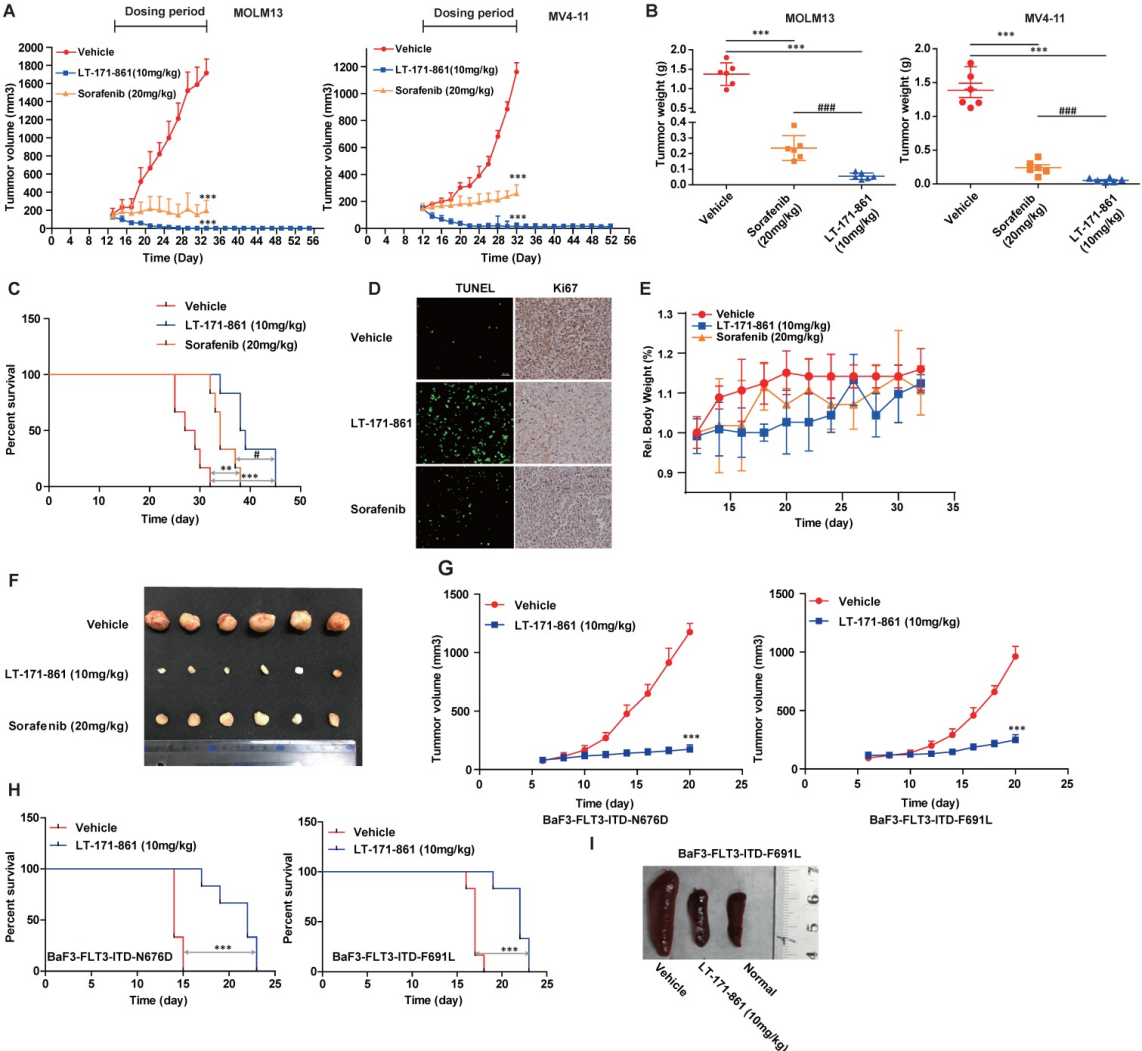
** Anti-leukemia activity of LT-171-861 in xenograft and engrafted mouse model.** (**A**) Mice (n=6) received LT-171-861, sorafenib and vehicle every two days for around 24 days. The group of mice treated with LT-171-861 (10 mg/kg) was subsequently monitored for an additional 20 days to examine tumor regrowth (ANOVA with Dunnett's posttest was performed, ***P< 0.001, **P< 0.01, *P< 0.05). Data were shown as mean±SD. (**B**) Representative photographs of tumors in each group after LT-171-861 (10 mg/kg), sorafenib (20 mg/kg) and the vehicle treatment. Comparison of the final tumor weights in each group after the treatment period (ANOVA with Dunnett's posttest was performed, ***P< 0.001, **P< 0.01, *P< 0.05, * represents control vs LT-171-861 and sorafenib, # represents LT-171-861 vs sorafenib). Data were shown as mean±SD. (**C**) An engrafted tumor model was used to evaluate *in vivo* antitumor activity. The NOD/SCID mice were intravenously inoculated with MV4-11 cells. From 12 days after inoculation, mice (n=6) received LT-171-861 (10 mg/kg) every other day. Log-rank testing was used to compare survival curves among treatment groups. LT-171-861 treatment resulted in significant survival advantage (***P< 0.001, **P< 0.01, *P< 0.05; * represents control vs LT-171-861 and sorafenib, # represents LT-171-861 vs sorafenib). (**D**) Apoptotic assays in engraft model were evaluated by TUNEL assay and cell proliferative activity was measured by Ki67 assay. Images were acquired at ×40 magnification. (**E**) BALB/c nude mice were subcutaneously inoculated with MV4-11 cells (5×10^6^) and bodyweight of each mouse was recorded every other day. (**F**) Representative photo of tumors from BALB/c nude mice which were inoculated with MV4-11 cells. (**G**) BALB/c nude mice were subcutaneously inoculated with BaF3 cells bearing FLT3-ITD-N676D (n=6) and FLT3-ITD-F691L (n=6) respectively. We administered 10 mg/kg LT-171-861 or vehicle once every other day. (**H**) BALB/c mice were intravenously inoculated with cells used in (G). Mice were treated with LT-171-861 (10 mg/kg) or vehicle once every other day. Log-rank testing was used to compare survival curves among treatment groups. LT-171-861 treatment resulted in significant survival advantage (***P< 0.001, **P< 0.01, *P< 0.05; * represents control vs LT-171-861). (**I**) Representative photos of spleens dissected from mice in (H) inoculated with BaF3 cells bearing FLT-3-ITD-F691L.

To measure the efficacy of LT-171-861 *in vivo*, we used two animal models including MOLM13- and MV4-11-xenograft and bone marrow engraftment mouse models. According to the results of PK/PD assays (Supplementary Table S1), an intravenous injection was selected to test the *in vivo* anti-tumor activity of LT-171-861. LT-171-861 was administered every two days in xenograft mice. Significant inhibition of tumor growth was observed in the LT-171-861-treated group (Figure 5A; Figure S3A). Remarkable inhibition of tumor growth and drastic tumor regression was observed in two xenograft models after 20 or 24-day treatment. Tumor-eliminated mice were monitored for an additional 20 days after dosing was halted. No tumor regrowth was observed in the LT-171-861-treated group, suggesting that complete tumor regression was achieved. Tumor weight and representative photos of tumors indicated that LT-171-861 had more potent antitumor activity than sorafenib did *in vivo* (Figure 5B and 5F). Furthermore, Ki67 and TUNEL assays were used to evaluate the anti-proliferative and pro-apoptotic activities (Figure 5D) of LT-171-861 *in vivo*. During the period of dosing, the bodyweight of every mouse was recorded every other day, and no significant weight suppression was observed among the three groups in MV4-11 cell line xenograft model (Figure 5E). At the end of the experiment, we performed H&E staining in the liver, heart, spleen, lung and kidney from the MV4-11 cell line xenograft model (Figure S3B), which showed no significant damage in these organs.

We next confirmed the anti-tumor activity of LT-171-861 on bone marrow engraftment, which is physiologically different from the xenograft model. Mice that received vehicle treatment died within 30 days after intravenous injection of MV4-11 cells. All mice in the LT-171-861- and sorafenib-treated groups had increased survival rates in comparison. Importantly, LT-171-861 showed superior efficacy to sorafenib (Figure 5C). Consistent with the data from the *in vitro* cell line experiments (Figure 2D), LT‑171-861 displayed antiproliferative activity on MV4-11 cells *in vivo*.

Finally, treatment with 10 mg/kg LT-171-861 every other day for 16 days inhibited tumor growth in subcutaneous xenograft models with BaF3 cells transfected with FLT3-ITD-N676D (Figure G, left) and FLT3-ITD-F691L (Figure G, right). The activity of LT-171-861 was also investigated in mice intravenously implanted with BaF3-transfected cells. Cells were injected into the tail vein of BALB/c nude mice and allowed to engraft for 5 days. Mice were dosed with either vehicle or 10 mg/kg LT-171-861 once a day followed by a 1-day break. This schedule was repeated for 8 cycles. Results showed that animals treated with LT-171-861 had a significant survival advantage (Figure 5H). Representative photos of the spleen from animals injected with BaF3-transfected cells demonstrated that treatment with LT-171-861 reduced the infiltration of BaF3-transfected cells in the spleen (Figure 5I).

In summary, two AML cell lines (MOLM13 and MV4-11) and two types of transfected BaF3 cells were used in xenograft and subcutaneous mouse models. These data demonstrated that LT-171-861 could be useful for the treatment of AML cells bearing FLT3-ITD mutations and several FLT3-ITD-TKD mutations.

### Effect of LT-171-861 against blasts from patients with AML

**Figure 6:**
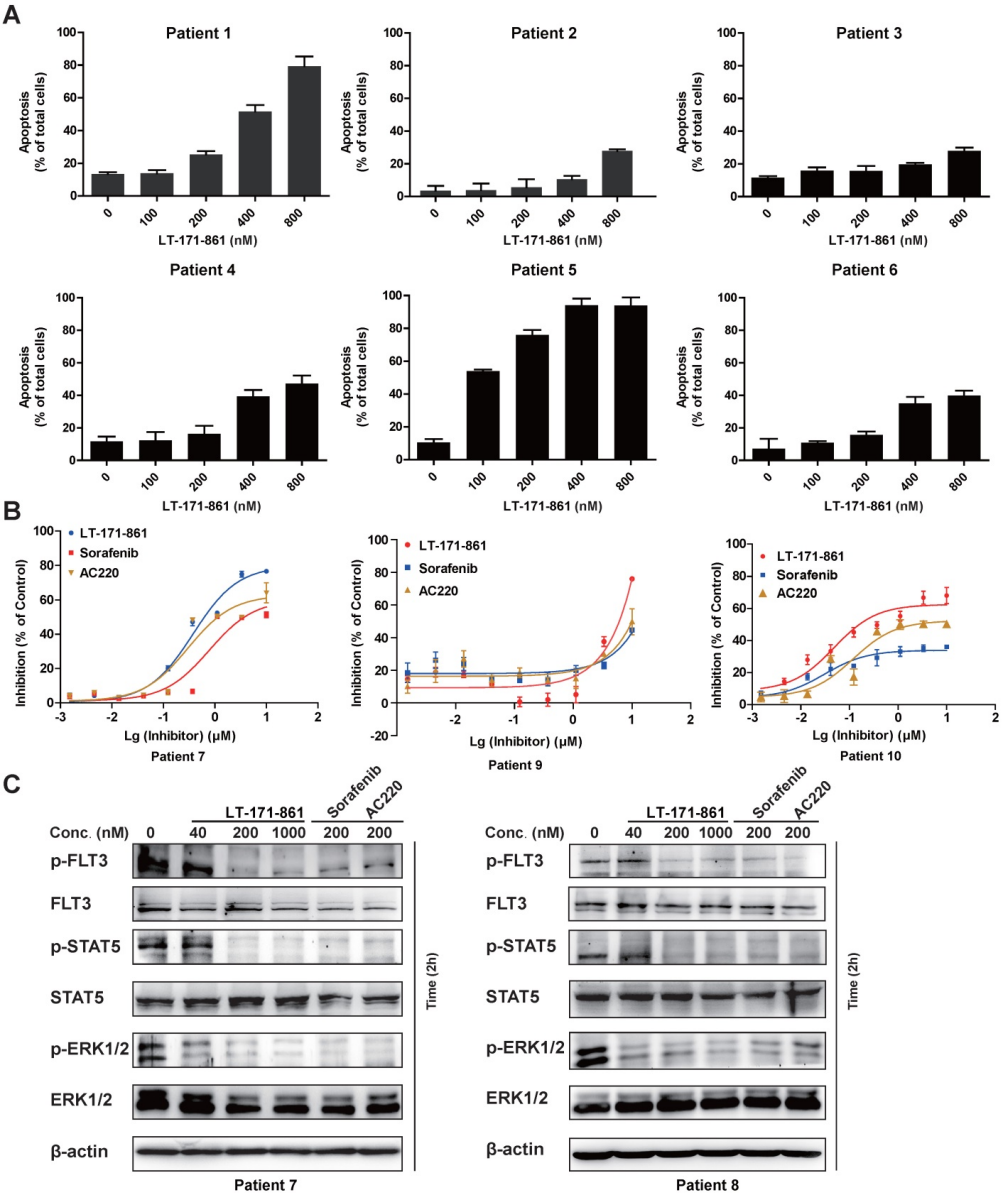
** Inhibitory efficacy of LT-171-861 against patient blasts.** (**A**) AML blasts harboring FLT3-ITD (patient 1 and 5) and FLT3-wt (patient 2-4 and 6) were treated with indicated concentrations of LT-171-861 for 36 h. Cells then stained with propidium iodide (PI)/annexin V and then evaluated by flow cytometry. (**B**) AML patient blasts expressing FLT3-ITD (patient 7, 9 and 10) were incubated with indicated concentrations of LT-171-861, sorafenib and AC220 for 72 h and cell viabilities were evaluated with MTT assay. (**C**) Blasts from AML patients harboring FLT3-ITD (patient 7 and 8) were incubated with LT-171-861 for 2 h, and p-FLT3, p-STAT5 and p-ERK1/2 were tested by western blot.

To evaluate antileukemia activity in primary cells, mononuclear cells from 10 AML patients diagnosed as FLT3-wild type (patient 2-4 and 6) and FLT3-ITD (patient 1 and 5) were collected. The results showed that AML cells with FLT3-ITD mutations were more sensitive to LT-171-861 treatment (Figure 6A). Cell viability assay demonstrated that LT-171-861 had comparable antileukemic activity with that of AC220 and sorafenib in blasts with FLT3-ITD mutations (Figure 6B). The levels of p-FLT3, p-STAT5 and p-ERK1/2 were also evaluated in blasts from patients with FLT3-ITD mutations (patient 7 and 8) by Western blot (Figure 6C).

In conclusion, our results in this report demonstrated that LT-171-861 is a highly potent FLT3 inhibitor in AML cells harboring FLT3-ITD mutation.

## Discussion

We showed that LT-171-861 has potent activity against AML cells and patient blasts with different FLT3 mutations, as well as in drug-resistant environments such as a stroma-protecting setting. LT-171-861 displayed potent inhibitory effect on both FLT3‑ITD mutations as well as on the known F691L, N676D, Y842C and D835Y mutations. Therefore, we focused on the therapeutic potential of LT-171-861 in FLT3‑mutant AML. Kinase profiling demonstrated an inhibition pattern for LT-171-861. We found that the IC_50_ values of LT-171-861 against FLT3 and its mutants were lower than those against other kinases in previous assays. We postulate that inhibition of FLT3 by LT-171-861 at such a low concentration as used in our study may be important for leukemia treatment. We show the potent efficacy of LT-171-861 against different FLT3 mutations (Figure 3). Other kinases such as FLT1, FLT4 and JAK are also known to be important downstream of FLT3, which may be why LT-171-861 shows superior effects against FLT3 even in a stroma-protecting environment 39, 40. As such, further studies with alternative targets are warranted.

Although many FLT3 inhibitors have been evaluated, issues such as poor PK/PD properties, marrow toxicities and drug resistance have become apparent in clinical trials. It has been proposed that FLT3 inhibitors often have activity against the structurally related receptor c-Kit 17, 24, which has important clinical implications as c-Kit is essential for normal hematopoiesis. Inhibition of this receptor can result in marrow suppression. MLN-518, midostaurin and quizartinib (AC220) are known to inhibit c‑Kit^14^, ^41^ , and marrow suppression has been shown in clinical trials. As shown in Table 1, the IC_50_ of LT-171-861 against c-Kit (1.81 μM) is 10000-fold higher than for FLT3 (0.234 nM). Furthermore, in our PK study, we showed that the bioavailability of LT-171-861 is around 15%, and its C_max_ is 0.20 μM (88.01 ng/mL), which is higher than CEP-701 and MLN-518 16. The oral pharmacokinetics of LT-171-861 are similar to PKC-412 (0.77 μM), which is the first approved agent for the treatment of AML with FLT3-ITD mutations 21. We also showed that the C_max_ of LT-171-861 was 50.06 μM, which is near 50,000-fold higher than the IC_50_ for FLT3-ITD inhibition in cellular assays within 2 h. Although the plasma half-life was short following both intravenous and oral administration, the plasma concentration of LT-171-861 remained around 50-fold (i.v) and 30 -fold (p.o) higher than the IC_50_ for FLT3-ITD inhibition in cellular assays after 12 h. According to a previous study from Bhagwat et al 16, after 24 h the plasma concentration of PKC-412 was lower than the IC_50_ for cellular assays. Together, these data show that the pharmacokinetic properties of LT-171-861 are more favorable than PKC-412, CEP-701 and MLN-518.

FLT3 is one of the most well-studied targets in the treatment of AML, and many candidate drugs are in clinical trials. Drug resistance remains a challenging problem, and acquired drug resistance to target therapies tends to occur, partially as a result of TKD mutations. FLT3-TKD mutations at N676, F691, Y842 and D835 were identified in patients with AML that conferred resistance to sorafenib and AC220 42-44. In this study, we demonstrated that LT-171-861 had inhibitory activity against FLT3‑ITD/D835Y, FLT3-ITD/F691L, FLT3-ITD/N676D and FLT3-ITD/Y842C mutants. Compared with AC220, LT-171-861 could still inhibit the proliferation of cells bearing ITD-F691L mutations. Moreover, the IC_50_ of LT-171-861 was near 16-fold lower than that of sorafenib in cells bearing ITD-D835Y mutations. Considering that BaF3 cell models have limitations in predicting clinical responses due to the heterogeneity of AML, we used primary cells from patients harboring FLT3-ITD mutations. LT-171-861 demonstrated potent pro-apoptotic activity correlating with FLT3 inhibition. Therefore, LT-171-861 is expected to inhibit FLT3 mutants conferring resistance to other TKIs in clinical studies.

In conclusion, we confirmed that LT-171-861 is a promising FLT3 inhibitor, and elucidation of the underlying mechanisms and a fuller understanding of LT-171-861 activity are required in future studies.

## Conclusions

In summary, we present a preclinical evaluation of LT-171-861 in AML cells with various FLT3 mutations. We report *in vitro* anti-leukemic activity of LT-171-861 that is as potent as that of AC220 and sorafenib in AML cells with FLT3-ITD mutations. In addition, LT-171-861 showed inhibitory efficacy against AML cells more rapidly than AC220 and sorafenib. Furthermore, LT-171-861 was able to overcome known drug resistance induced by FLT3-ITD/F691L and FLT3-ITD/D835Y mutations, which limit the efficacy of AC220 and sorafenib in patients with AML.

We also showed that LT-171-861 had modest bioavailability with high plasma concentration in *in vivo* models; therefore, further studies are needed to optimize its bioavailability. In two animal models of AML, we found that LT-171-861 is useful for the treatment of AML cells with FLT3-ITD mutations and several FLT3‑ITD-TKD mutations. Together, our data suggest that LT-171-861 may be an effective therapeutic strategy in patients with FLT3-ITD/F691LAML- and FLT3-ITD/D835Y-mutant AML. Our research also provides insight into the potential targets and mechanisms of LT-171-861, and potential LT-171-861 drug combinations.

## Supplementary Material

Supplementary figures and tables.

## Figures and Tables

**Table 1:** Kinase profile of LT-171-861

Kinase	Compound IC50* (M)	IC50 (M) Control compound	Control compound ID
	LT-171-861		
c-Kit	1.81E-06	1.04E-07	Staurosporine
c-Src	7.20E-08	1.76E-09	Staurosporine
ERK1	1.06E-06	4.90E-09	SCH772984
ERK2/MAPK1	7.98E-07	6.34E-10	SCH772984
FGFR1	2.00E-08	4.04E-09	Staurosporine
FLT1/VEGFR1	5.60E-09	8.41E-09	Staurosporine
FLT3	2.34E-10	1.08E-09	Staurosporine
FLT3 (D835Y)	1.32E-10	9.19E-11	Staurosporine
FLT3 (ITD)	7.18E-10	1.26E-09	Staurosporine
FLT4/VEGFR3	1.24E-09	1.62E-09	Staurosporine
FMS	2.47E-07	1.70E-09	Staurosporine
JAK1	5.50E-08	6.95E-10	Staurosporine
JAK2	3.38E-08	1.02E-10	Staurosporine
JAK3	3.26E-08	1.32E-10	Staurosporine
PIM2	2.06E-06	4.67E-08	Staurosporine

LT-171-861 was tested in 10-dose IC_50_ mode with a 3-fold serial dilution starting at 1 or 10 μM. Control compound, staurosporine, was tested in 10-dose IC_50_ mode with 40-fold serial dilution starting at 20 μM. Alternate control compounds were tested in 10-dose. IC_50_ mode with 4-fold serial dilution starting at 10 μM. Reactions were carried out at 10 μM ATP.

**Table 2:** Potency comparison of FLT3 inhibitors in cellular assays

Compound	Human leukemia cell line (IC_50_, nM)				BaF3 cells with FLT3 mutants IC_50_ (nM)					
	MV4-11	MOLM13	THP-1	U937	ITD	D835Y	ITD-D835Y	ITD-F691L	ITD-Y842C	ITD-N676D
LT-171-861	1.8	1.3	504.5	495.4	4.1	23.3	57.2	7.65	18.8	47.5
AC220	1.5	1.2	>1000	>1000	9.2	31.2	55.3	100.9	43.4	212.4
Sorafenib	3.2	5.3	>1000	>1000	13.2	235.4	878.3	45.2	346.5	103.2

For evaluation of IC_50_ values in human Leukemia cell lines with FLT3-ITD mutation, MV4-11 and MOLM13 cells were treated with test inhibitors and cell viability was measured. The BaF3 model cell expressing different FLT3 mutations, FLT3-ITD, FLT3-D835Y, FLT3-ITD/D835Y, FLT3-ITD/F61L, FLT3-ITD/Y842C, and FLT3-ITD/N676D, were treated with test inhibitors, and cell viability was measured.

## Abbreviations

AML: Acute myeloid leukemia
FLT3: Fms-like tyrosine receptor kinase 3
ITD: internal tandem duplication
TKD: Tyrosine kinase domain
CR: complete remission
IC_50_: Half maximal inhibitory concentration
C_max_: Maximum concentration
CETSA: cellular thermal shift assay
PK: Pharmacokinetics
PD: Pharmacodynamics
CM: Conditional medium
NM: Normal medium
BMMCs: Bone marrow mononuclear cells
PBMCs: Peripheral blood mononuclear cells
