# Supplementary material for: LT-171-861, a novel FLT3 inhibitor, shows excellent preclinical efficacy for the treatment of FLT3 mutant acute myeloid leukemia
Source: Theranostics. 2021 Jan 1;11(1):93–106. doi: 10.7150/thno.46593 (PMC7681098; doi:10.7150/thno.46593)
Supplement: Supplementary file 1 — Supplementary figures and tables. [file thnov11p0093s1.pdf]

Supplementary Materials

LT-171-861, a novel FLT3 inhibitor, shows excellent preclinical efficacy for the treatment of acute myeloid leukemia

Zhou Yu,[#] Jiaying Du,[#] Hui Hui,[#] Shaoxin Kan, Tongxin Huo, Kai Zhao, Tao Wu, Qinglong Guo, and Na Lu

State Key Laboratory of Natural Medicines, Jiangsu Key Laboratory of Carcinogenesis and Intervention, School of Basic Medicine and Clinical Pharmacy, China Pharmaceutical University, 24 Tongjiaxiang, Nanjing 210009, People's Republic of China;

[#]Zhou Yu, Jiaying Du and Hui Hui contributed equally to this study

Additional information

Correspondence: Na Lu

State Key Laboratory of Natural Medicines, Jiangsu Key Laboratory of Carcinogenesis and Intervention, School of Basic Medicine and Clinical Pharmacy, China Pharmaceutical University, 24 Tongjiaxiang, Nanjing 210009, People's Republic of China

E-mail: nalu@cpu.edu.cn

Supplementary Table S1. Oral and intravenous pharmacokinetics parameters of LT-171-861

Parameters	Units	IV	IG
$AUC_{(0-t)}$	ng/mL*h	3314.72	462.62
$AUC_{(0-\infty)}$	ng/mL*h	3377.66	530.62
$MRT_{(0-t)}$	h	1.86	4.28
$MRT_{(0-\infty)}$	h	2.1	5.95
t1/2z	h	2.02	3.68
T_{max}	h	0.25	1
CLz	mL/h/kg	2960.63	18845.72
Vz	mL/kg	8612.54	100186.3
C_{max}	ng/mL	2167.5	88.01

Supplementary Table S2. Clinical data for patients with AML

Patient no.	Diagnosis	Source	Blasts initial PB%	FLT3 status	Status
1	AML	PB	78%	ITD	New
2	AML	PB	54%	wt	New
3	AML	PB	63%	wt	New
4	AML	PB	71%	wt	New
5	AML	PB	42%	ITD	New
6	AML	PB	58%	wt	New
7	AML	PB	67%	ITD	Relapse
8	AML	PB	45%	ITD	Relapse
9	AML	PB	86%	ITD	Relapse
10	AML	PB	87%	ITD	Relapse

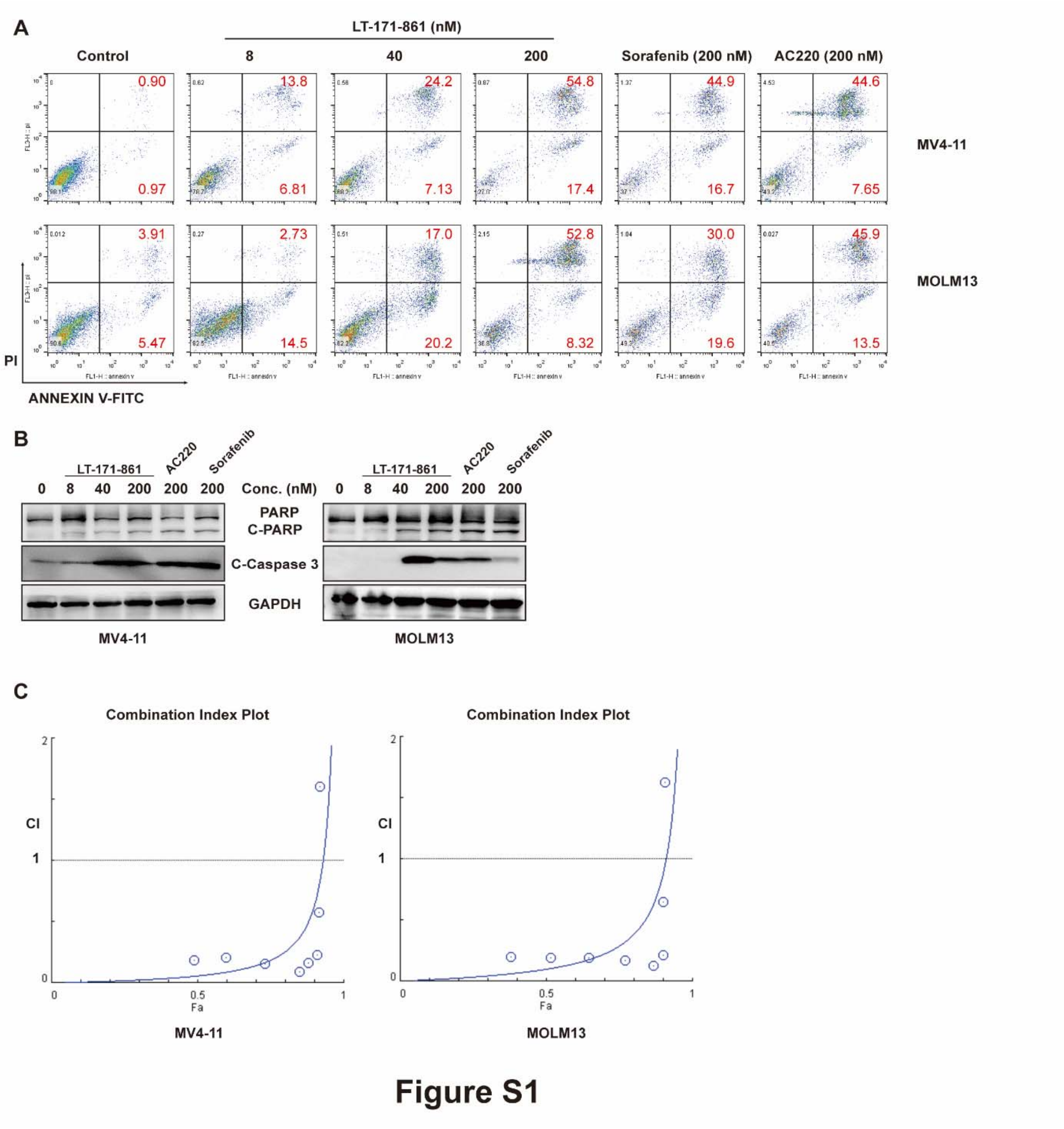

Supplementary Figure S1. Pro-apoptotic effects of LT-171-861 and established FLT3 inhibitors on leukemia cell lines expressing FLT3-ITD

(A) MV4-11 and MOLM-13 cells treated with indicated concentrations of LT-171-861, AC220 or sorafenib respectively for 36 h were stained by Annexin V-FITC and PI. Then flow cytometry assay was used to evaluate the ratio of dead cell. (B) MV4-11 and MOLM-13 cells were incubated with LT-171-861, AC220 or sorafenib at indicated concentrations for 36 h. PARP, and cleaved caspase-3 were evaluated in the cell lysate by western blot. (C) Combination index (CI) value calculated by Compusyn2.0 software.

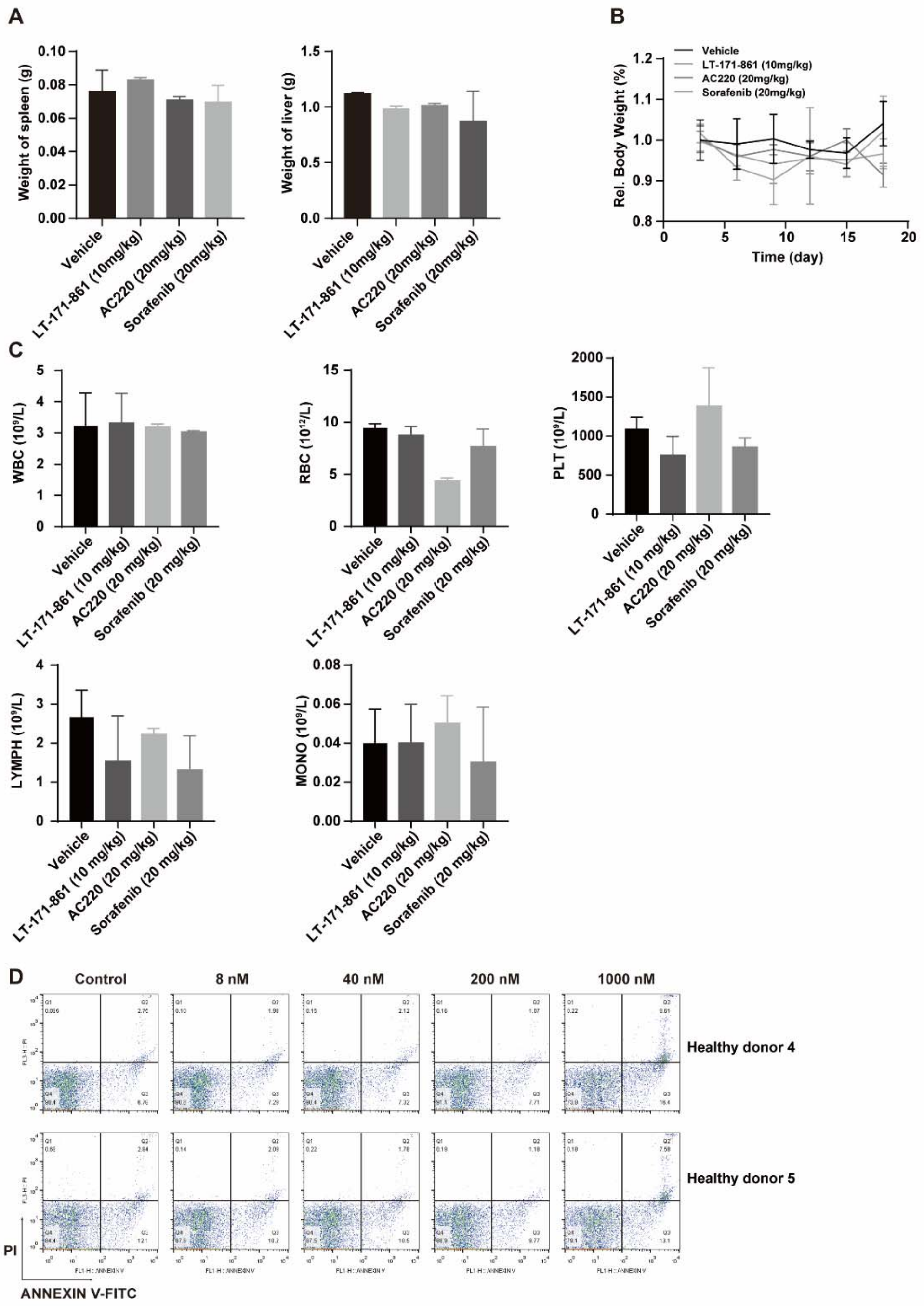
A
Weight of spleen (g)
Weight of liver (g)
Vehicle
LT-171-861 (10mg/kg)
AC220 (20mg/kg)
Sorafenib (20mg/kg)
B
Rel. Body Weight (%)
Time (day)
C
WBC (10⁹/L)
RBC (10¹²/L)
PLT (10⁹/L)
LYMPH (10⁹/L)
MONO (10⁹/L)
LT-171-861 (10 mg/kg)
AC220 (20 mg/kg)
Sorafenib (20 mg/kg)
D
Control
8 nM
40 nM
200 nM
1000 nM
Healthy donor 4
Healthy donor 5
PI
ANNEXIN V-FITC

Figure S2

Supplementary Figure S2. Toxicity evaluation of LT-171-861 in C57BL/6 mice and normal PBMCs from healthy donors.

Healthy C57BL/6 mice (n=6) were intravenously injected with vehicle or LT-171-861 (10 mg/kg) every other day and were orally administered with AC220 (20 mg/kg) or sorafenib (20 mg/kg) once per day for 18 days. 24 h after the last administration, weights of spleens and livers (A), relative body weights (B), and total peripheral blood evaluations (C) populations were analysed. WBC=white blood cells; RBC=red blood cells; PLT=platelets; LYMPH=lymphocytes; MONO=monocytes. Means $\pm$ SD are shown. (D) PBMCs isolated by Ficoll from healthy donors were incubated with indicated concentrations of LT-171-861 for 36 h. Cells then stained with Annexin V-FITC/PI and evaluated by flow cytometry. Annexin V^+/PI^- and Annexin V^+/PI^+ cells were considered dead cells.

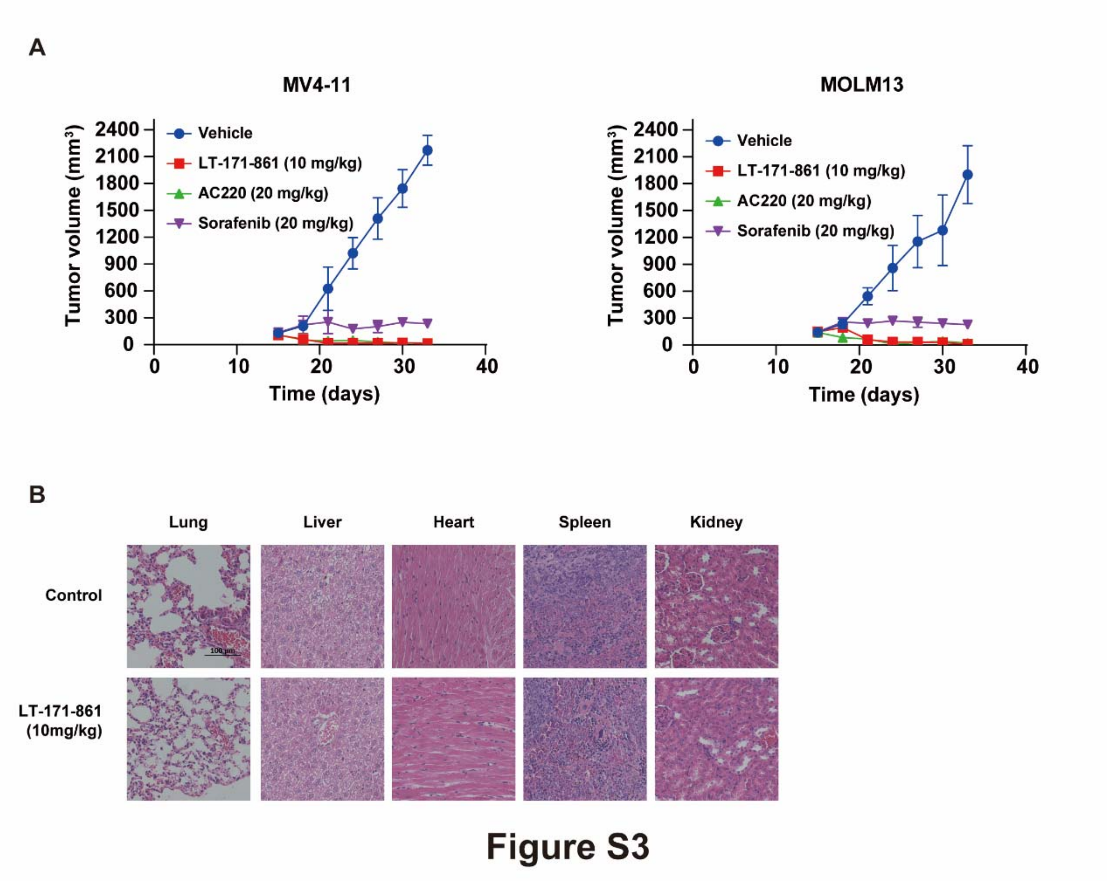

Supplementary Figure S3. Anti-leukemia activity of LT-171-861 and established FLT3 inhibitors AC220 and sorafenib in xenograft mouse model.

(A)Mice (n=6) administered with LT-171-861 intravenously every two days and with AC220 (20 mg/kg) or sorafenib (20 mg/kg) orally once a day for 21 days. Tumor volumes were measured by caliper. Mean±SD were shown. (B) Mice were sacrificed when they exhibited hind-limb paralysis after LT-171-861 treatment, and their tissues were resected and fixed in 4% formalin for HE analysis. Images were acquired at ×20 magnification.
